# Exploring the link between particulate matter pollution and acute respiratory infection risk in children using generalized estimating equations analysis: a robust statistical approach

**DOI:** 10.1186/s12940-024-01049-3

**Published:** 2024-01-25

**Authors:** Mihir Adhikary, Piyasa Mal, Nandita Saikia

**Affiliations:** Department of Public Health and Mortality Studies, International Institute for Population Sciences, Mumbai, Maharashtra India

**Keywords:** Air pollution, Particulate matter, Acute respiratory infection, Child ARI, Air pollution in India, Pollution and health

## Abstract

**Background:**

India is facing a burdensome public health challenge due to air pollution, with a particularly high burden of acute respiratory infections (ARI) among children. To address this issue, our study aims to evaluate the association between exposure to fine particulate matter (PM_2.5_) and ARI incidence in young children in India.

**Materials and methods:**

Our study used PM_2.5_ data provided by the Atmospheric Composition Analysis Group at Washington University to assess the association between PM_2.5_ exposure and ARI incidence in 223,375 children sampled from the 2019–2021 Demographic Health Survey in India. We employed the generalized estimating equation and reported odds ratios and 95% confidence intervals for a 10 µg/m^3^ increase in PM_2.5_ and quartiles of PM_2.5_ exposure.

**Results:**

Each 10 µg/m^3^ increase in PM_2.5_ levels was associated with an increased odds of ARI (OR: 1.23, 95% CI: 1.19–1.27). A change from the first quartile of PM_2.5_ (2.5–34.4 µg/m^3^) to the second quartile (34.5–51.5 µg/m^3^) of PM_2.5_ was associated with a two-fold change (OR: 2.06, 95% CI: 1.60–2.66) in the odds of developing ARI. Similarly, comparing the first quartile to the fourth quartile of PM_2.5_ exposure (78.3–128.9 µg/m^3^) resulted in an over four-fold increase in the odds of ARI (OR: 4.45, 95% CI: 3.37–5.87).

**Conclusion:**

Mitigation efforts must be continued implementing higher restrictions in India and to bring new interventions to ensure safe levels of air for reducing the burden of disease and mortality associated with air pollution in India.

## Background

Air pollution has become a major public health concern worldwide, with particulate matter (PM) pollution being one of the most significant contributors to adverse health outcomes. PM pollution is a complex mixture of solid and liquid particles suspended in the air with a diameter of less than 10 micrometres (PM_10_) or less than 2.5 micrometres (PM_2.5_). Exposure to PM pollution can lead to several adverse health effects, including respiratory and cardiovascular diseases, stroke, and lung cancer [1–3]. Children are particularly vulnerable to the health effects of PM pollution, with exposure to PM pollution having harmful effects on their developing respiratory and immune systems [4].

### Biological mechanisms underlying the association between PM pollution and ARI in children

There are several biological mechanisms that have been proposed to explain the association between PM pollution and ARI in children. PM pollution contains a complex mixture of particles of various sizes and compositions that can have different effects on the respiratory system. One of the proposed mechanisms is that PM pollution can directly damage the respiratory tract and impair the function of the airway cells [5]. PM pollution can cause oxidative stress and inflammation in the respiratory tract, which can damage the respiratory epithelial cells and reduce their ability to clear pathogens and pollutants from the airways [5, 6]. This can lead to increased susceptibility to respiratory infections, including ARI. Another proposed mechanism is that PM pollution can impair the function of the immune system, particularly in children with immature immune systems [7]. Exposure to PM pollution can increase the levels of pro-inflammatory cytokines and reduce the levels of anti-inflammatory cytokines in the respiratory tract, which can impair the immune response to respiratory infections. This can lead to increased susceptibility to ARI and more severe symptoms [7]. PM pollution can also exacerbate pre-existing respiratory conditions such as asthma [8–11] and allergies, which can increase the risk of ARI. Exposure to PM pollution can trigger asthma attacks and worsen asthma symptoms, making children more vulnerable to respiratory infections [8–11]. Additionally, exposure to PM pollution can worsen allergic reactions, which can increase the risk of ARI. Finally, PM pollution can also facilitate the transmission of respiratory infections by increasing the survival and transmission of respiratory pathogens in the air [12]. PM pollution can act as a carrier for respiratory viruses and bacteria, allowing them to travel longer distances and remain suspended in the air for longer periods of time [12]. This can increase the likelihood of transmission of respiratory infections to susceptible individuals, including children.

### Fine PM pollution and ARI in children: epidemiological evidence

Epidemiological studies have investigated the relationship between PM pollution and ARI in children, and most of these studies have found a positive association between exposure to PM pollution and increased risk of ARI in children [4]. A study conducted in China found that children exposed to higher levels of PM_2.5_ were at a higher risk of developing ARI compared to those exposed to lower levels [13]. A meta-analysis of 35 studies conducted worldwide found a significant positive association between PM pollution and ARI in children [14]. The analysis showed that exposure to PM pollution increased the risk of lower respiratory illness by approximately 15%. This association was found to be consistent across different geographic regions, age groups, and study designs. There is also evidence suggesting that exposure to PM pollution can worsen the severity of ARI in children. A study conducted in China found that exposure to higher levels of PM_2.5_ was associated with longer hospital stays and higher healthcare costs for children with ARI [15]. Overall, the epidemiological evidence strongly supports the association between PM pollution and ARI in children, with several studies providing consistent evidence of this association across different populations and settings.

India is one of the countries with the highest burden of acute respiratory infections (ARI) among children globally [16]. The country is also known for having high levels of particulate matter (PM) pollution, primarily from sources such as vehicular emissions, industrial activities, and biomass burning [17]. Several studies have investigated the relationship between PM pollution and ARI among children in India [18]. For example, a study conducted in Delhi found that exposure to PM_2.5_ pollution was associated with an increased risk of ARI among children [18–20]. Another study conducted in Ahmedabad city of western India found that high levels of PM_2.5_ pollution were associated with an increased risk of ARI among children [21].

In 2016, chronic respiratory disease (COPD) accounted for 6.4% of the total disability-adjusted life years (DALYs) in India, up from 4.5% in 1990. Air pollution was responsible for 53.7% (43.1–65) of the COPD-related DALYs in India in 2016 [16]. While previous studies in India have primarily examined the impact of solid-fuel use on the health of young children, few have focused on PM_2.5_ as the primary exposure. Although studies that have investigated exposure to PM_10_ have demonstrated a positive correlation between PM_10_ exposure and respiratory symptoms in children in India [4, 22], research on PM_2.5_ exposure and its effects is limited. Despite these promising findings, there are still significant gaps in our understanding of the impact of PM pollution on ARI among children in India. Further research is needed to better understand the whole mechanisms underlying this association and to evaluate the effectiveness of public health interventions aimed at reducing exposure to PM pollution Therefore, we conducted an analysis to assess the association between PM_2.5_ exposure and acute respiratory infections (ARI) in young children in India.

The findings of this study will have critical implications for public health interventions aimed at reducing the burden of ARI among children in India. By identifying the association between PM_2.5_ pollution and ARI, this study can inform the development of targeted interventions to reduce exposure to PM pollution and prevent and treat ARI in children.

In summary, this study aims to contribute to the growing body of research on the impact of fine PM pollution on children’s health, particularly in the context of ARI. The findings of this study will be relevant not only to India but also to other low- and middle-income countries where the burden of ARI and PM pollution is high. This research can inform evidence-based policies and interventions to improve child health outcomes and reduce the overall burden of disease.

## Materials and methods

### Data and sample

This study analyzed data from the fifth round of the Indian version of Demographic Health Survey (DHS), called as National Family Health Survey (NFHS-5). NFHS-5 fieldwork was done in two phases: phase one from 17 June 2019 to 30 January 2020 and phase two from 2 January 2020 to 30 April 2021 in all the states (28 states) and union territories (8 UTs). The survey was carried out utilizing a two-stage stratified random sampling technique and the sample was drawn from the primary sampling units (PSUs); the PSUs consist both the rural and urban areas (classified as Census Enumeration Blocks). The NFHS-5 collected data from 724,115 women and 101,879 men while the individual response rate for males was 92%, and for women, it was 97%. Further the survey provides information on 232,920 children under the age 5 years along with the details of 636,699 household characteristics such as the type of cooking fuel used with a response rate of 98%. The NFHS-5 was developed to be representative of each of India’s over 707 districts for key indicators, including child health outcomes. The detailed methodology for the survey design can be found in the NFHS-5 national report [23]. Out of a total of 232,920 children in the dataset, 8675 observations were missing for the primary outcome variable, leaving us with a final sample size of 223,375.

### Outcome variable

The outcome variable of this study is acute respiratory infections among children under five years of age. The survey acquired this information based on symptoms experienced by children under five years of age two weeks preceding the survey [23]. The survey used two questions to collect this information: the first question asked whether the child had experienced a cough in the past two weeks, while the second question asked whether the child had experienced shortness of breath, rapid breathing, or difficulty breathing when they had a cough. In the National Family Health Survey-5 (NFHS-5), ARI was defined as a cough accompanied by chest-related symptoms such as shortness of breath or rapid breathing. For analysis in this study, ARI was dichotomized into two categories: “yes” (coded as 1) for children who had experienced coughing or difficulty breathing in the past two weeks, and “no” (coded as 0) for those who had not experienced any symptoms of ARI during that time. This definition has been used in previous studies to assess the prevalence of ARI among young children [24–28].

### Exposure variable

We used global annual surface levels of PM_2.5_ estimates for 2020 provided by the Atmospheric Composition Analysis Group at Washington University. The estimates were obtained using aerosol optical depth (AOD) measurements from various satellite products, including NASA MODIS C6.1, MISR v23, MAIAC C6, and SeaWiFS satellite data [29]. The data was then combined and related with surface levels of PM_2.5_ and AOD using geographically weighted regression (GWR). The resulting estimates were assessed at a resolution of 0.01° × 0.01° (approximately 1 km × 1 km) for the exposure assessment. The dataset is publicly available on the following website: https://sites.wustl.edu/acag/datasets/surface-pm2-5/. To assess levels of PM_2.5_ in India in 2020, we used geographic data from the Standard DHS dataset for India in 2019–2021, which included geocode data. We extracted the mean PM_2.5_ level from each cluster in the dataset and used it as the primary exposure variable. Our study found that levels of PM_2.5_ in India varied significantly between clusters.

### Confounders and adjustments

We have tried to identify the potential confounders which could affect the outcome when analysing the association between level of PM_2.5_ and ARI symptom. The identification of confounders was based on a thorough review of existing literature and theoretical frameworks which establishes the relationship between particulate matter pollution and ARI symptom among children [30–32]. We considered variables that had been previously established as potential sources of bias in similar studies and we decided the following confounders to keep in our model- Current age of child (continuous: years), Sex of Child (dichotomous: male/female), Highest educational level of mother (categorical: No education, Primary, Secondary, and Higher), Household wealth quintile (categorical: Poorest, Poorer, Middle, Richer, Richest), Type of place of residence (dichotomous: Rural/Urban), Mother’s Smoking habit (Smokes cigarettes/tobacco and Does not smoke), Type of cooking fuel used in the HH (clean fuel and unclean fuel). To calculate the wealth index, principal component analysis was conducted on two sets of variables: ownership of consumer goods (such as television, bicycle, and car etc.) and conditions of living space (such as access to drinking water, toilet facilities, and flooring materials etc.) [23]. We divided home fuel consumption into two categories: (1) clean fuels which includes electricity, LPG, natural gas, and biogas, and (2) unclean fuels include kerosene, coal and lignite, biomass (including charcoal, wood, straw/shrubs/grass, agricultural residues, animal manure).

### Statistical analysis

We used descriptive statistics to show the characteristics of study population and bivariate cross tabulations to calculate the prevalence of ARI symptom by different socio-demographic characteristics of the study population. The differences were then assessed using chi square statistics and the p values were shown. Since our exposure variable values are same within the clusters, we also nested the outcome variable within clusters. Then we used generalized estimating equations (GEE) analysis to assess the association between the exposure to PM_2.5_ level and prevalence of ARI. GEE models are commonly used in epidemiology, social sciences, and other fields where data are collected over time or clustered by location or other grouping factors [33–35]. So, we used GEE for adjusting the effects of clustering on overall outcomes. The goal behind applying GEE is to make inferences about the population when accounting for the within-subject correlation. For every one-unit increase in a covariate across the population, GEE tells us how much the average response would change. In the analysis of clustered data, researchers commonly employ mixed-effect or multilevel models. However, in our approach, we utilized Generalized Estimating Equations (GEE), which fundamentally differs as it constitutes a marginal model. GEE aims to model the population average, in contrast to mixed-effect or multilevel models, which are characterized as subject-specific or conditional models. The latter models (mixed-effect or multilevel) enable the estimation of distinct parameters for each subject or cluster, signifying that the parameter estimates are contingent upon the specific subject or cluster. This (GEE), consequently, affords valuable insights into the variability among subjects or clusters. During the whole analysis we appropriately specified the survey design by using ‘svyset’ command in STATA software.

We estimated the crude/unadjusted association between PM_2.5_ and ARI in two different ways. The first one shows increase in prevalence of ARI with 10 unit increase in the level of PM_2.5_ and in the second one, we categorized PM_2.5_ into quartiles to see how a certain level of PM_2.5_ associated with ARI. Then we have incorporated the confounding variables in our adjusted model, and we have reported the adjusted odds ratios. We have two different adjusted model for GEE in the study since we have tried to see the association of PM_2.5_ with ARI in two different ways as mentioned earlier, in first one (model 2) we consider PM_2.5_ as a continuous variable and in second one (model 2) we divided PM_2.5_ into quartiles. To assess the shape of the association and identify potential nonlinearities in the relationship we utilized restricted cubic spline analysis with three knots for PM_2.5_. The three knots were placed at predetermined percentiles of the PM_2.5_ distribution, which helped to ensure that the model was adequately flexible to capture the true shape of the relationship. By examining the results of the spline analysis, we were able to gain insights into the nature of the relationship between PM_2.5_ and the risk of ARI, which can inform public health interventions and policies aimed at reducing exposure to air pollution. The whole statistical analysis was done on STATA MP version 14.0 [36] and the maps were created on ArcMap version 10.0.

## Results

### Sample characteristics and ARI prevalence

Table 1 shows the sample distribution of the study population (*n* = 223,375) and the prevalence of ARI among children. As shown, 115,381 (51.7%) of the children were male and 107,994 (48.4%) were female. Overall, 48,436 (21.68) mothers don’t have any formal education, 28,653 (12.83%) have primary, 115,339 (51.6%) have secondary and 30,949 (13.9%) have higher educational qualifications. Most mothers do not smoke, i.e., 210,865 (94.4%) and live in rural areas, i.e., 177,984 (79.6%). Overall, 127,456 (57.1%) households used uncleaned fuel as a source of energy for cooking, while 95,919 (42.9%) households used cleaner fuels (electricity, liquefied petroleum gas, natural gas, biogas and solar).

**Table 1:** Characteristics of study population and the prevalence of ARI symptom among the children in India, 2019–2021

Background characteristics	Sample	Children with ARI symptom % (95% CI)	P value
**PM2.5 quartiles**			
Q1 (2.5–34.4 µg/ m^3^)	25.34 (56,601)	1.96 (1.78–2.17)	*P* < 0.001
Q2 (34.5–51.5 µg/m^3^)	25.15 (56,188)	2.39 (2.17–2.63)	
Q3 (51.6–78.2 µg/m^3^)	24.87 (55,552)	2.74 (2.54–2.96)	
Q4 (78.3–128.9 µg/m^3^)	24.64 (55,034)	3.59 (3.36–3.84)	
**Current age of child**	***Mean ± SD***	***2.03 ± 1.43***	
0	19.99 (44,656)	3.34 (3.11–3.58)	*P* < 0.001
1	19.37 (43,278)	3.31 (3.07–3.57)	
2	19.70 (44,007)	2.72 (2.52–2.93)	
3	19.91 (44,482)	2.51 (2.31–2.71)	
4	21.02 (46,952)	2.11 (1.94–2.31)	
**Sex of child**			*P* < 0.001
Male	51.65 (115,381)	3.02 (2.87–3.18)	
Female	48.35 (107,994)	2.54 (2.41–2.68)	
**Highest educational level of mother**			*P* < 0.001
No education	21.68 (48,436)	2.87 (2.66–3.10)	
Primary	12.83 (28,653)	3.38 (3.08–3.70)	
Secondary	51.63 (115,339)	2.81 (2.66–2.96)	
Higher	13.85 (30,947)	2.17 (1.96–2.39)	
**Household wealth quintile**			*P* < 0.001
Poorest	26.90 (60,077)	3.2 (2.99–3.43)	
Poorer	23.30 (52,044)	3.07 (2.86–3.30)	
Middle	19.47 (43,485)	2.69 (2.48–2.92)	
Richer	16.93 (37,807)	2.41 (2.19–2.65)	
Richest	13.41 (29,962)	2.35 (2.12–2.60)	
**Type of place of residence**			*P* < 0.001
Urban	20.32 (45,391)	2.3 (2.08–2.53)	
Rural	79.68 (177,984)	2.97 (2.84–3.10)	
**Mother’s smoking habit**			*P* < 0.001
Smokes cigarettes/tobacco	5.60 (12,510)	4.13 (3.58–4.76)	
Does not smoke	94.4 (210,865)	2.75 (2.64–2.87)	
**Type of cooking fuel used in the HH**			*P* < 0.001
Clean fuel	42.94 (95,919)	2.59 (2.44–2.76)	
Unclean fuel	57.06 (127,456)	2.97 (2.82–3.12)	
**Total (n)**	**223,375**	**2.78 (2.68–2.91)**	

Children residing in PM_2.5_ first, second, third and fourth quartiles reported ARI symptoms were 1109 (1.96%), 1343 (2.39%), 1522 (2.74%) and 1976 (3.59%), respectively. ARI was reported for 3485 (3.02%) among male and 2743 (2.54%) among females. Overall, 1922 (3.2%) children from the poorest, 1598 (3.07%) from the poorer, 1170 (2.69%) from the middle, 911 (2.41%) from the richer and 672 (2.17%) from the richest household were suffering from ARI. In rural areas, 5286 (2.97%) children reported ARI; in urban areas, it was 1044 (2.3%). ARI was reported for 3785 (2.97%) children when uncleaned fuel was used for cooking and 2484 (2.59%) when cleaned fuel was used as a source of energy for cooking. Overall, 517 (4.13%) children reported ARI if mothers had a smoking habit; if mothers did not smoke, it was 5799 (2.75%).

### The concentration of PM_2.5_ and district-level ARI

**Fig. 1:**
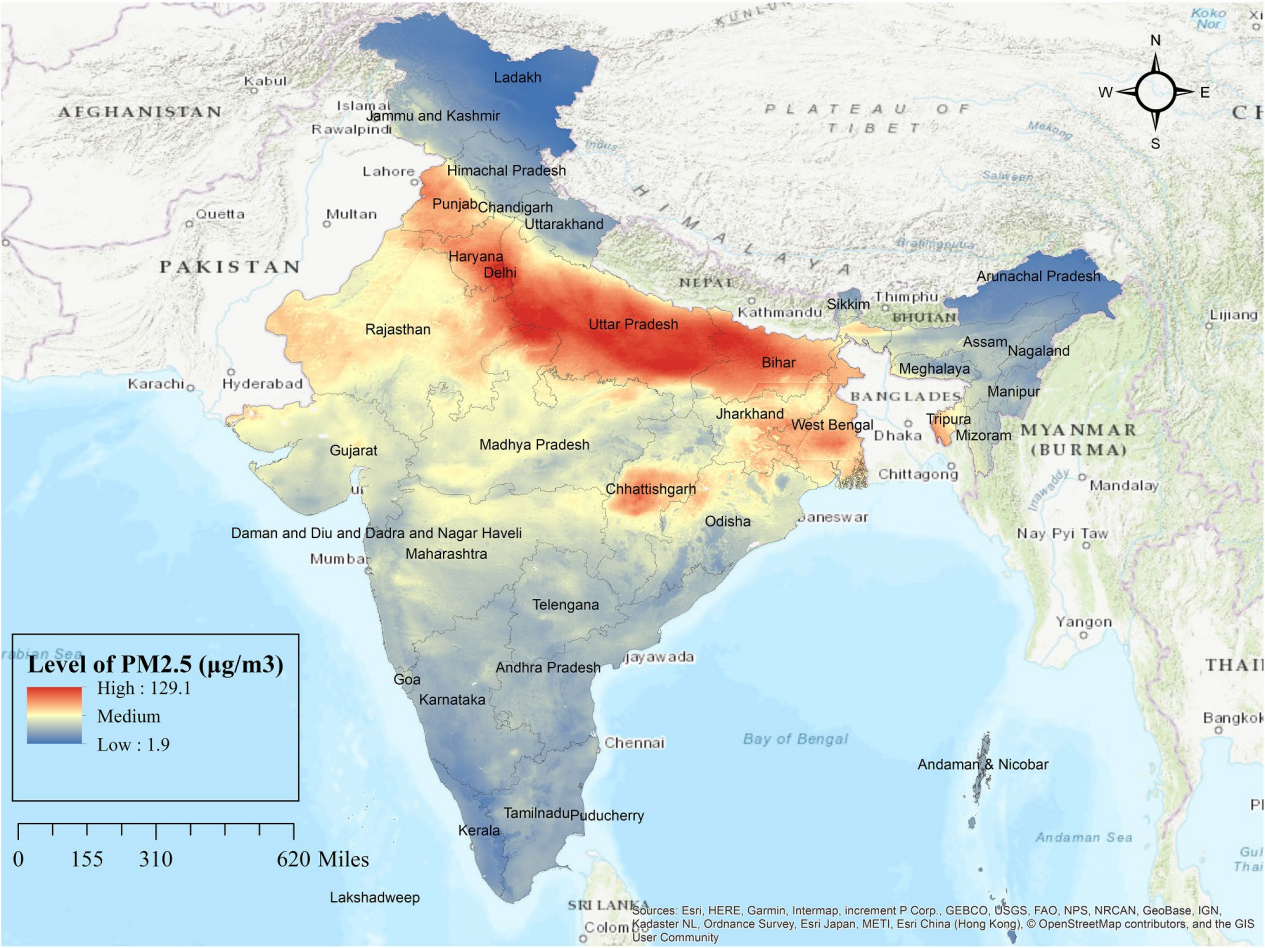
Level of PM_2.5_ in India (2020)

**Fig. 2:**
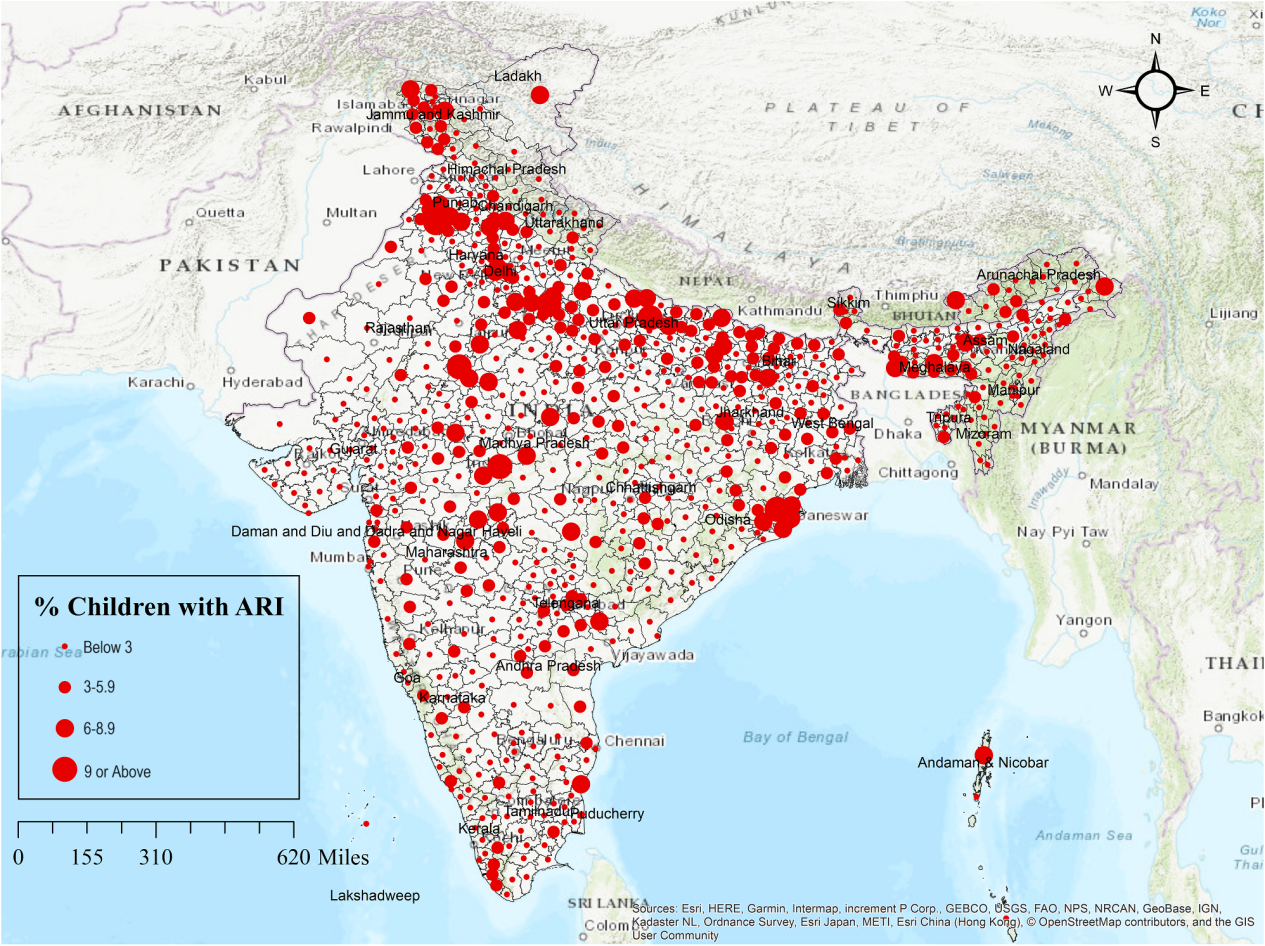
Prevalence of ARI symptoms among children under age 5 in the districts of India (2019–2021)

Figure 1 shows the level of PM_2.5_ in India in 2020 and Fig. 2 shows the prevalence of ARI among children under the age of 5 in India (2019–2021). The level of PM_2.5_ is highest in Delhi. It is very high in the north Indian plain, followed by West Bengal and part of Chhattisgarh. It is moderate in central India and relatively low in the southern part of India, Uttarakhand, Himachal and Northeast India. The level of PM_2.5_ is lowest in Kashmir, Ladakh, Arunachal Pradesh and part of Kerala. In India, in almost 1% of districts, more than 9% of children reported ARI. These are districts of Delhi, and part of Odisha. Whereas in 7% of the district, 6 to 8.9% of children reported ARI. These districts are mainly situated in the north Indian plain, Meghalaya, part of Madhya Pradesh. Most of these mentioned districts are highly polluted by PM_2.5_. On the other hand, in 67% district, less than 3% of children reported ARI and these districts are comparatively less polluted by PM_2.5_.

### Association between PM_2.5_ and ARI among children

Table 2 summarizes the results of Generalized estimating equations (GEE) analysis showing the association between PM_2.5_ exposure and ARI in children under one model that involves no covariates adjustment and another two models adjusted for various traits. In the unadjusted/crude analysis, the result shows 10 µg/m^3^ increase in total mass annual PM_2.5_ was associated with greater odds of ARI in children [OR: 1.222; CI: 1.180, 1.261]. After categorizing PM_2.5_ in four quartiles, ARI symptoms are almost two times [OR: 1.989; CI: 1.544, 2.563] higher in third quartile PM_2.5_ and more than four times [OR: 4.219; CI: 3.202, 5.560] higher in fourth quartile PM_2.5_, than first quartile of PM_2.5_. However, the increasing ARI in the second PM_2.5_ quartile became insignificant.

**Table 2:** Generalized estimating equation (GEE) results showing the association between PM_2.5_ pollution and ARI in children

Background characteristics	Crude OR [CI]	Adjusted OR [CI]	
		Model-1	Model-2
**PM**_**2.5**_ **(every 10 µg increase/m** ^**3**^ **)**	1.222*** (1.018, 1.0261)	1.231*** (1.192, 1.270)	-
**PM**_**2.5**_ **Quartiles**			
Q1 (2.5–34.4 µg/m^3^)	1 (1, 1)	-	1 (1, 1)
Q2 (34.5–51.5 µg/m^3^)	1.12 (0.900, 1.404)	-	1.131 (0.904, 1.415)
Q3 (51.6–78.2 µg/m^3^)	1.989*** (1.544, 2.563)	-	2.067*** (1.604, 2.662)
Q4 (78.3–128.9 µg/m^3^)	4.219*** (3.202, 5.560)	-	4.450*** (3.374, 5.870)
**Current age of child**		0.991 (0.971, 1.012)	0.99 (0.970, 1.011)
**Sex of child**			
Male		1 (1, 1)	1 (1, 1)
Female		0.925** (0.871, 0.981)	0.925* (0.872, 0.982)
**Mother’s highest educational level**			
No education		1 (1, 1)	1 (1, 1)
Primary		1.236* (1.042, 1.465)	1.246* (1.050, 1.479)
Secondary		1.036 (0.894, 1.200)	1.039 (0.897, 1.203)
Higher		0.946 (0.777, 1.152)	0.933 (0.767, 1.134)
**Wealth quintile**			
Poorest		1 (1, 1)	1 (1, 1)
Poorer		1.099 (0.952, 1.269)	1.089 (0.944, 1.258)
Middle		0.961 (0.800, 1.154)	0.953 (0.793, 1.144)
Richer		0.811 (0.653, 1.007)	0.806 (0.649, 1.001)
Richest		0.745* (0.573, 0.969)	0.764* (0.589, 0.990)
**Type of place of residence**			
Urban		1 (1, 1)	1 (1, 1)
Rural		1.311* (1.031, 1.667)	1.284* (1.009, 1.633)
**Mother’s smoking habit**			
Smokes cigarettes/tobacco		1 (1, 1)	1 (1, 1)
Does not smoke		0.484*** (0.367, 0.638)	0.487*** (0.369, 0.642)
**Type of cooking fuel used in the HH**			
Clean fuel		1 (1, 1)	1 (1, 1)
Unclean fuel		0.807** (0.702, 0.926)	0.813** (0.709, 0.933)
***Cons.***	4.400*** (3.559, 5.439)	8.157*** (5.268, 12.631)	16.9*** (10.942, 26.104)

*p < 0.05, **p < 0.01, ***p < 0.001

This association between PM_2.5_ and ARI persisted in the adjusted models. In adjusted model 1, after adjusting age and sex of the child, mother’s educational status, household wealth quintile, type of place of residence, mother’s smoking habit, and type of cooking fuel used in the household, there is a significant positive association [OR: 1.231; CI: 1.192, 1.270] between PM_2.5_ exposure (every 10 µg increase/m^3^) and ARI in children. Apart from PM_2.5_, the sex of the child, type of place of residence, mother’s smoking habit, and type of cooking fuel used in the household are also significant modifying factors for ARI in children. After adjusting other background variables, female children have a lower odds than male children of having ARI, which is inconsistence with previous findings. Children living in rural areas have a higher odds of having ARI than urban areas, and mothers who don’t have smoking habits have lower odds of having ARI than mothers who have. Children living in households that use uncleaned fuel have lower odds of ARI, which is inconsistent with previous findings. In adjusted model-2, the background variables showed a similar kind of association as adjusted model-1.

**Fig. 3:**
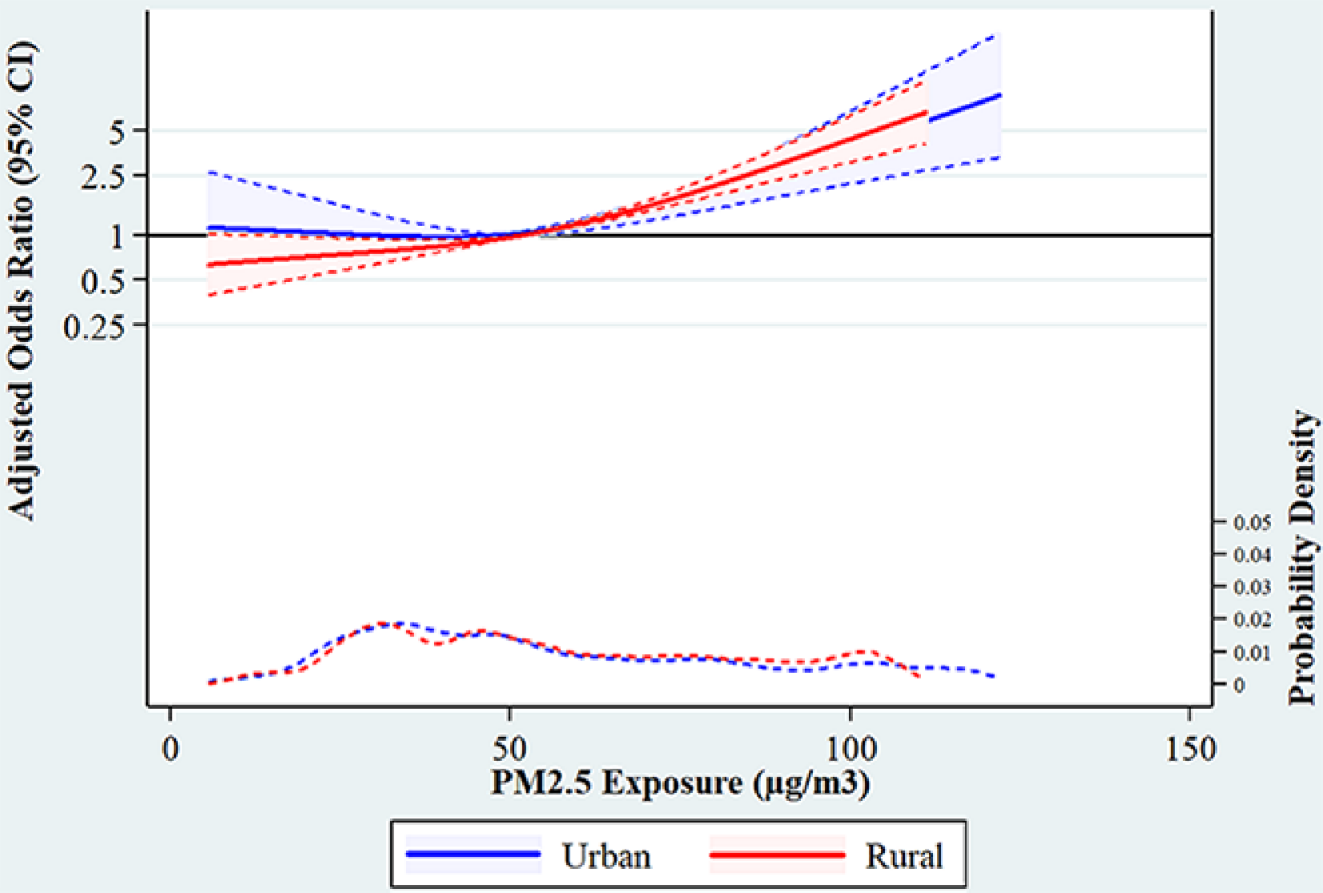
Restricted cubic Spline plot showing the association between exposure to PM2.5 and the prevalence of ARI among children and in India adjusted for age, sex of child, mother’s education, mothers smoking status, cooking fuel, wealth index and rural-urban residence

Figure 3 shows the restricted cubic spline as a smooth function to model the non-linear relationship between exposure to PM_2.5_ and the prevalence of ARI among children. Model is adjusted for age, sex of child, mother’s education, mothers smoking status, cooking fuel, wealth quintile. Adjusted odds of ARI among children has increased with increasing level of PM_2.5_ in rural area. However, in urban area, up to the level of 50 µg/m^3^ of PM_2.5_ concentration the odds of ARI are 1, after that it gradually increased with increasing level of PM_2.5_.

## Discussion

Our study shows that PM_2.5_ pollution is significantly associated with children’s acute respiratory system. There are a considerable number of environmental and medical pieces of literature about the mechanism of how PM_2.5_ affects the respiratory system [37–39]. Compared to larger particles, PM_2.5_ penetrates the airways more deeply, and some of the particles can enter the bloodstream and cause systemic effects [37, 40]. Children breathe more quickly than adults do; as a result, they inhale more contaminants. Moreover, their respiratory systems are still developing. When children inhale PM_2.5_, these particles can reach deep into the lungs, causing irritation and inflammation that limits airflow, making breathing difficult for children and leading to coughing and wheezing [41]. Long-term exposure to high levels of PM_2.5_ can weaken the immune system and increase the risk of asthma, chronic bronchitis, pneumonia, and reduced lung function [31, 42, 43].

Our study found that ARI is more among male children. An explanation may be that boys are more likely to play outside where they are more in contact with pollution, which raises their risk of respiratory illnesses. A biological explanation is that boys generally have smaller airways than girls of the same age and female lungs mature earlier [44].

Mothers’ smoking habits are one of the causes of ARI among children. Earlier studies while investigating the association of maternal smoking and acute respiratory infection symptoms, also found the similar kind of results [45–47].

After adjusting other factors, our study shows that uncleaned fuel reduces the ARI among children. This is inconsistence with previous studies. Previous studies show that children living in homes where uncleaned fuel was used for cooking had a greater probability of acute respiratory infections among children as it is related to indoor air pollution [48, 49]. Although descriptive statistics show in household where uncleaned fuel is used, children are more prone to ARI. An explanation may be that in the polluted area, PM_2.5_ is more effective factor than uncleaned fuel for ARI among children. Recent studies shows that ambient air pollution erodes the benefit of using clean fuel in India [50].

From spatial analysis, it is observed that in most areas where PM_2.5_ pollution is very high, children have reported ARI, for example, the north Indian plain. But in the North-eastern region, where PM_2.5_ pollution is very low, for instance, Meghalaya, Assam, a considerable percentage of children has reported ARI. This may be due to uncleaned fuel and an attached kitchen to the living room. According to NFHS 5, about 63.4% and 56.4% of household used uncleaned fuel as a source of cooking fuel in Meghalaya and Assam, respectively [23].

From the restricted cubic spline, our study found that up to 50 PM_2.5_, children are not suffering from ARI. After that level, it increases dramatically. In 10 PM_2.5_ levels, ARI prevalence among children is almost five times that of children residing in 50 PM_2.5_. These findings support the National Ambient Air Quality Standards (NAAQS), where 40 PM_2.5_ is the threshold value of PM_2.5_ in India.

This study shows that children living in rural areas are more prone to acute respiratory systems, although urban areas are more polluted than a rural area. While investigating the association between PM_2.5_ and ARI among children in 35 developing countries, a previous study explained that socio-economic variations such as clean fuel and access to healthcare services; and spatiotemporal differences in air pollutants regarding source and composition between urban and rural areas are responsible for that [30].

### Strength and limitation

Our study had significant advantages, including the use of a large, nationally representative survey with a high response rate of 98%. Furthermore, we found that the use of solid-fuel did not influence the link between PM_2.5_ exposure levels and acute respiratory infection in children, which was a significant factor in previous studies. However, there are some limitations to our research. First, the measurement of PM_2.5_ levels was at the cluster level, which may have led to exposure misclassification and potential bias. Nevertheless, this misclassification is likely to be non-differential and move the effect towards the null. Second, the health outcomes for ARI were self-reported, and maternal recall bias may have affected their accuracy. It is worth noting that the responses on ARI were recalled from two weeks before the survey, indicating that maternal recall bias is unlikely to have had a significant impact. Furthermore, previous research suggests that this type of bias is non-differential. However, there were still several limitations to our study, including the inability to establish a causal relationship due to the cross-sectional study design. Additionally, we found that PM_2.5_ levels were exceedingly high throughout India, which may have led to an underestimation of the impact of PM_2.5_ on ARI outcomes. Additionally, we might not able to control every crucial factors (confounders) that may modify the risk factor due to data restriction.

## Conclusion

In summary, our research indicates that exposure to high levels of PM_2.5_ increases the odds of developing ARI in children under five years old. Therefore, it is imperative that India continues to implement effective mitigation strategies and enforces stricter air quality regulations to ensure safe air for all, especially vulnerable groups such as pregnant women, older adults, and children. Mitigation efforts must be continued implementing higher restrictions in India and to bring new interventions to ensure safe levels of air for reducing the burden of disease and mortality associated with air pollution in India.

## Data Availability

The datasets generated and/or analysed during the current study are available in the DHS program repository: https://dhsprogram.com/methodology/survey/survey-display-541.cfm. The PM_2.5_ dataset is publicly available on the following website: https://sites.wustl.edu/acag/datasets/surface-pm2-5/.
